# Lap Welding of Nickel-Plated Steel and Copper Sheets Using Coaxial Laser Beams

**DOI:** 10.3390/ma18143407

**Published:** 2025-07-21

**Authors:** Kuan-Wei Su, Yi-Hsuan Chen, Hung-Yang Chu, Ren-Kae Shiue

**Affiliations:** Department of Materials Science and Engineering, National Taiwan University, Taipei 106, Taiwan; r11527037@ntu.edu.tw (K.-W.S.); d13527010@ntu.edu.tw (Y.-H.C.); f11527008@ntu.edu.tw (H.-Y.C.)

**Keywords:** blue-light laser, coaxial laser welding, heterogeneous lap joint, copper, nickel-plated steel, microstructure

## Abstract

The laser heterogeneous lap welding of nickel-plated steel and Cu sheets has been investigated in this study. The YAG (Yttrium-Aluminum-Garnet) laser beam only penetrates the upper Ni-plated steel sheet and cannot weld the bottom Cu sheet due to the low absorption coefficient of the YAG laser beam. Incorporating a blue-light and fiber laser into the coaxial laser beam significantly improves the quality of the weld fusion zone. The fiber laser beam can penetrate the upper nickel-plated steel sheet, and the blue-light laser beam can melt the bottom copper sheet. Introducing the blue-light laser to the coaxial laser beams overcomes the low reflectivity of the bottom copper sheet. The fiber/blue-light coaxial laser continuous welding can achieve the best integrity and defect-free welding. It shows potential in the mass production of the next generation of lithium batteries.

## 1. Introduction

Lithium-ion power batteries are rechargeable batteries with high power and high energy density output [1]. They are commonly used in applications such as electric vehicles, power tools, and drones requiring high energy density, repeated charge and discharge cycles, and low self-discharge rates [2,3,4,5,6]. The positive electrode materials of lithium-ion batteries mainly include lithium cobalt oxide (LiCoO_2_), lithium manganese oxide (LiMn_2_O_4_), lithium nickel oxide (LiNiO_2_), and lithium iron phosphate [7,8]. Here, aluminum foil is often used as the current collection layer of the positive electrode. The primary function of the negative electrode material is to store lithium ions. The more lithium ions it can store, the higher the energy density of the unit battery cell. The negative electrode material is mostly silicon and graphene [7,8]. Copper foil serves as the current collection layer, connecting the electrode to the external circuit. This copper foil is often used as the current-collecting layer of the negative electrode. When a lithium battery is charged, lithium ions are generated at the battery’s positive electrode and pass through the electrolyte to the negative electrode, embedding in the layered structure and micropores of the negative electrode material. The more lithium ions accumulate, the higher the charging capacity. When a lithium battery is discharged, the lithium ions embedded in the negative electrode material are released, returned to the positive electrode through the electrolyte, and gathered at the copper current collection layer.

The cylindrical lithium-ion battery is covered with a cylindrical nickel-plated steel can on the outside, which serves as the negative electrode grounding [9,10]. The thickness of the nickel-plated steel can is approximately 300 μm. The thickness of the copper current collecting layer is between 8 and 12 μm. The copper collecting layer at the bottom of the battery and the nickel-plated steel can are connected to form a current path. Most cylindrical lithium batteries in mass production are made using resistance welding, which forms a lap joint between the bottom of the nickel-plated steel can and the copper foil inside the battery [11,12,13,14]. The main disadvantage of resistance welding is that during mass production, the heterogeneous lap joint is prone to spattering metal droplets, empty welds, poor contact, and high joint electrical resistance. These problems urgently need to be improved to meet the demand for producing next-generation lithium batteries with higher energy density.

Laser welding is a joining process that uses a high-power laser beam as the heat source to melt alloys for welding [15,16,17,18,19,20]. It is a high-energy-density welding method since its energy density is as high as 10^6^ W/cm^2^ [21]. Compared to traditional medium and low-energy-density welding methods, laser welding has the advantages of low total heat input, reduced distortion of the base metal caused by heat, and the ability to obtain a high-quality, high-aspect-ratio weld [22]. Since different metals have very different absorption coefficients for laser light sources of different wavelengths, this absorption rate significantly impacts the weld’s quality [23,24,25]. Therefore, selecting the appropriate laser beam for welding is essential based on the characteristics of various metals.

**Figure 1:**
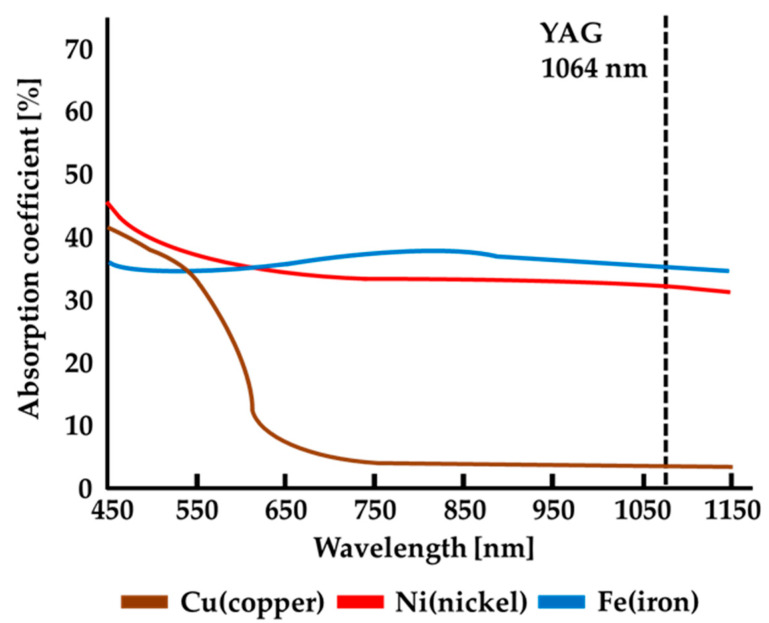
Absorption coefficients of three metals for laser beams of different wavelengths.

Figure 1 shows the absorption coefficients of three metals, copper, nickel, and iron, for laser beams of different wavelengths [26]. The absorption coefficients of laser beams of different wavelengths for nickel and iron are pretty good (approximately between 30% and 45%). Using lasers of various wavelengths in the figure is sufficient for laser welding the Ni-plated steel plate. In contrast, the copper plate has a significantly low absorption coefficient (only about 2%) for traditional YAG lasers with a wavelength of 1064 nm [27,28,29]. This means that if a laser beam with this wavelength is used for copper welding, not only will the laser beam not be effectively absorbed by the copper metal surface to form a weld, but it will also be reflected or scattered by the copper metal surface. In severe cases, it may even cause damage to the laser lens, so the copper metal welding is unsuitable for use with the YAG laser. However, a shorter wavelength of 450 nm blue-light laser is an appropriate heat source for copper welding due to its high absorption coefficient of 43%. In that case, the absorption coefficient of blue-light laser on the copper metal surface can be significantly increased to 43%, so laser welding of copper metal can be made appropriately.

There are many studies on the laser welding of copper using a blue-light laser [30,31,32,33,34,35,36]. However, the lap welded joint’s microstructure, element mixing, and phase distribution have not been studied in detail. This investigation aims to evaluate the feasibility of using coaxial laser beams to perform lap welding of nickel-plated steel and copper sheets. The Ni-plated steel sheet was used to simulate the steel can, and the copper sheet was used to simulate the current collection layer at the negative electrode of the cylindrical lithium battery. The cross-section of the heterogeneous lap weld was analyzed in greater depth to observe the effects of different combinations of laser parameters on the weld microstructures.

## 2. Materials and Experimental

A YAG laser (Neodymium-doped YAG, Coherent, Taipei Taiwan) and the coaxial fiber/blue-light laser (TPLBDL450-500, Turning Point Lasers Corp., Hsinchu, Taiwan) beams performed the heterogeneous lap welding of Ni-plated steel and copper sheets. The laser beam was irradiated on the top surface of the Ni-plated steel sheet, penetrated the steel sheet, and welded the bottom Cu sheet to form a heterogeneous lap joint. The direction of the laser beam applied in welding was arranged to simulate the manufacturing of cylindrical lithium batteries in mass production.

**Figure 2:**
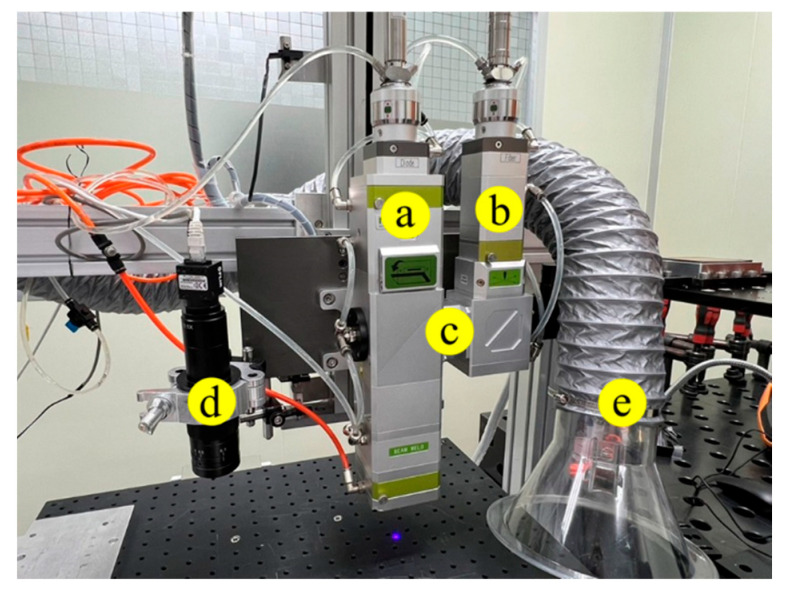
The infrastructure of the coaxial laser beams used in the experiment. (**a**) The blue-light laser beam, (**b**) the fiber laser beam, (**c**) the reflector lens to guide the fiber laser beam to the optical axis of the blue-light laser beam, (**d**) the red light laser beam for alignment, and (**e**) the air extraction pipe.

The experiment was divided into YAG laser spot lap welding, fiber/blue-light coaxial laser spot lap welding, and fiber/blue-light coaxial laser continuous lap welding. The wavelength of the YAG was 1064 nm, its pulse power was 500 W, the pulse width was 12 ms, and the beam diameter was 150 µm. The laser beam was focused 200 µm below the surface of the nickel-plated steel sheet. Fiber/blue-light coaxial laser welding was also performed in the experiment. The fiber laser beam first generated an amplified laser signal by stimulating radiation in the resonant cavity, then guided it to the lens group through the optical fiber, and finally guided the laser beam to the same optical axis as the blue-light laser beam. The spot size of the blue-light laser was 300 µm, and its wavelength was 450 nm. The spot size of the fiber laser was 50 µm, and its wavelength was 1080 nm. The fiber/blue-light coaxial laser beams were focused 200 µm below the surface of the nickel-plated steel sheet. An Ar shielding gas with a flow rate of 25 L/min was applied during welding to prevent the oxidation of the weld. The infrastructure of the coaxial laser beams used in the experiment is displayed in Figure 2.

The chemical composition of the steel plate in wt% was 0.118 C, 0.024 Cr, 0.015 Cu, 0.106 Mn, 0.025 Ni, 0.012 P, 0.009 S, 0.139 Si, and Fe balance. The steel sheet with a thickness of 300 μm was coated with a pure nickel layer with a thickness of 2 μm. The oxygen-free copper sheet with a thickness of 1 mm was used in the experiment. A fixture secured the nickel-plated steel and copper sheets to ensure they were fixed in place before laser lap welding. Table 1 shows all laser welding conditions used in the experiment.

The weld was cut with a slow-speed diamond saw to obtain its cross-section, mounted with epoxy resin, and then subjected to a standard metallographic procedure before analysis. The weld’s cross-section microstructure and chemical composition were analyzed using an electron probe microanalyzer (EPMA), JEOL JXA-8530F Plus (JEOL Co., Ltd., Tokyo, Japan). A JEOL JSM-7800F (JEOL Co., Ltd., Tokyo, Japan) field emission electron microscope (FESEM) and an electron backscatter diffraction (EBSD) instrument were used to analyze the crystal structure of the weld. The AztecCrystal software was applied to obtain crystallographic information such as the phase distribution, strain distribution, boundary misorientation angle distribution, and grain size.

## 3. Results and Discussion

### 3.1. Microstructural Analyses of the YAG Laser Spot Welding Specimen

**Figure 3:**
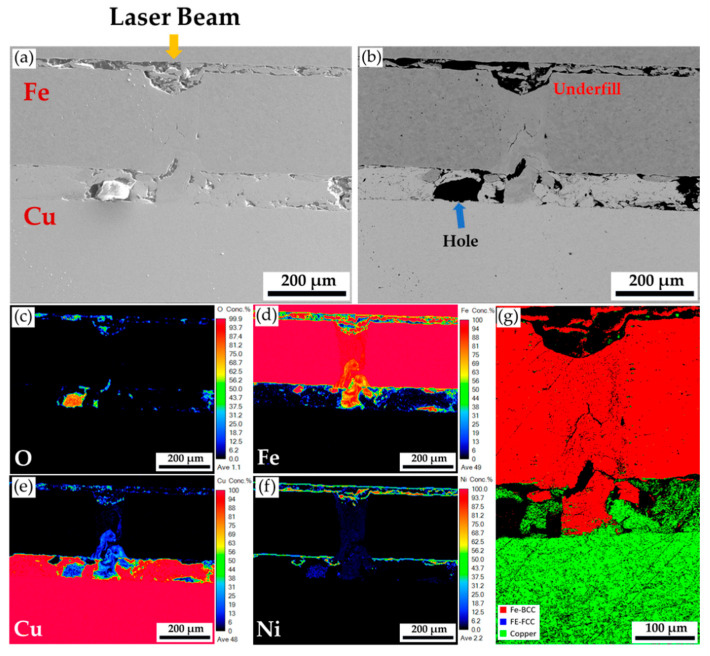
The cross-section of the YAG laser spot welding specimen: (**a**,**b**) EPMA SEI and BEI; (**c**–**f**) EPMA quantitative element mappings of O, Fe, Cu, and Ni in wt%; and (**g**) EBSD phase map

The YAG laser heterogeneous spot lap welding has been performed first for comparison purposes. Figure 3 shows the cross-section of the YAG laser spot welding specimen. Figure 3a,b displays secondary electron image (SEI) and backscatter electron image (BEI) of the weld. An underfilled cavity can be observed at the laser incident point of the top Ni-plated steel sheet, indicating that the laser beam energy is too high. Many cracks are also generated in the weld’s Ni-plated steel sheet. In addition, there is a delamination phenomenon and many crack holes between the Ni-plated steel and Cu sheets. The crack holes and delamination phenomenon are not only present at the weld but are also distributed between the two metal sheets. They are caused by the fact that the upper Ni-plated steel and lower copper sheets are not in tight contact after the laser beam is applied. The nickel-plated steel and copper sheets are closely pressed before YAG laser lap welding. The high-energy density laser beam generates metal vapor and plasma gas during welding. Because the absorption coefficient of the solid Cu to the 1060 μm YAG laser beam is extremely low (approximately 2%), the laser beam’s energy cannot penetrate the Cu sheet. Most of the YAG laser beam is reflected by the surface of the copper sheet, and it can only be scattered between the Ni-plated steel and the copper sheet. In Figure 3a,b, only the surface layer of the copper sheet is melted and solidified into a loose Cu-rich layer.

Figure 3c–f displays EPMA quantitative element mappings of O, Fe, Cu, and Ni in wt%, respectively. Minimal mixing between the upper Ni-plated steel and the lower Cu sheets is observed in these figures. There is a loosely resolidified Cu-rich layer and many holes between the two sheets. Since the Cu has a very high reflectivity to the YAG laser beam, stratification of the copper sheet occurs due to the resolidification of the copper surface. Figure 3c is a quantitative mapping of the oxygen element. The stratified holes are oxidized due to the absence of a protective Ar atmosphere. Figure 3g shows the phase map in the EBSD analysis. The dissimilar weld cannot be electrically polished because of the different polishing rates between the steel and Cu. When the automatic grinding polishing machine is applied, loose particles always fall onto the abrasive disk. Therefore, a few scratches remained after polishing. The BCC Fe (ferrite) is not mixed with the Cu after YAG laser welding, as illustrated in Figure 3g. A fusion zone with good integrity is not achieved between the nickel-plated steel and copper sheet. The low absorption coefficient of the YAG laser beam to the Cu sheet damages the possibility of forming an acceptable heterogeneous lap weld in the experiment. The application of the YAG laser is not appropriate in the current case.

### 3.2. Microstructural Analyses of the Fiber/Blue-Light Coaxial Laser Spot Welding Specimen

**Figure 4:**
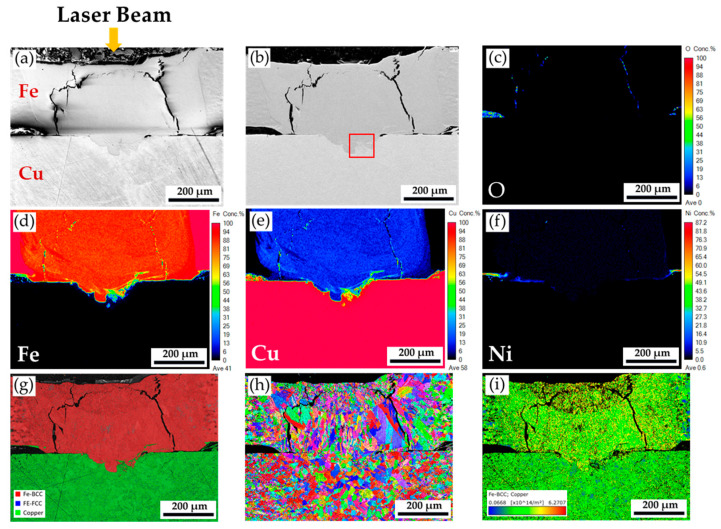
The cross-section of the fiber/blue-light coaxial laser spot welding specimen: (**a**,**b**) EPMA SEI and BEI; (**c**–**f**) EPMA quantitative element mappings of O, Fe, Cu, and Ni in wt%; EBSD (**g**) phase map, (**h**) IPF, and (**i**) GND density map.

Figure 4 shows the cross-section of the fiber/blue-light coaxial laser spot welding specimen. Figure 4a,b displays two long cracks in the Ni-plated steel sheet. It is inferred that the stress was too high when the weld was cut by a low-speed diamond saw, causing the cracks to initiate and propagate. The Ni-plated steel sheet is laser-welded to the Cu sheet, and a heterogeneous lap weld has been successfully formed. The penetration depth of the weld is 86 µm, and its width is 243 µm. The laser welding mode belongs to the conduction mode according to the depth-to-width ratio of the weld [21,22].

In Figure 4c, the weld exhibits almost no oxidation, including the top and interface of the two sheets. According to the quantitative element mappings of Fe and Cu illustrated in Figure 4d,e, approximately 10~20 wt% Cu is dissolved in Fe in the upper portion of the weld, and Fe is not in Cu. It is consistent with the Cu-Fe binary alloy phase diagram [37,38]. The solubility of Cu in austenite at high temperatures is significantly higher than that of Fe in Cu. Figure 4g shows some Cu phase in the weld fusion zone of the original bottom Ni-plated steel. It is deduced that part of the Cu plate is melted due to the blue-laser beam irradiation, but the energy density of the blue-laser beam is insufficient to make a convection of keyhole mode. Figure 4h shows the inverse pole figure (IPF) of the weld. Many coarse cellular grains are growing upward on the top of the Ni-plated steel weld, indicating the direction of the highest temperature gradient, i.e., the source of the coaxial laser beam. In Figure 4i, the geometrically necessary dislocation (GND) density map in the weld fusion zone is higher than in the steel and Cu base metals. However, the GND density map in the coaxial laser beam weld (Figure 4i) shows a uniform distribution. Lower residual stress is expected in the coaxial laser beam welding. It is evident that adding a blue-light laser beam to the coaxial laser beam welding greatly improves the quality of the heterogeneous lap weld.

**Figure 5:**
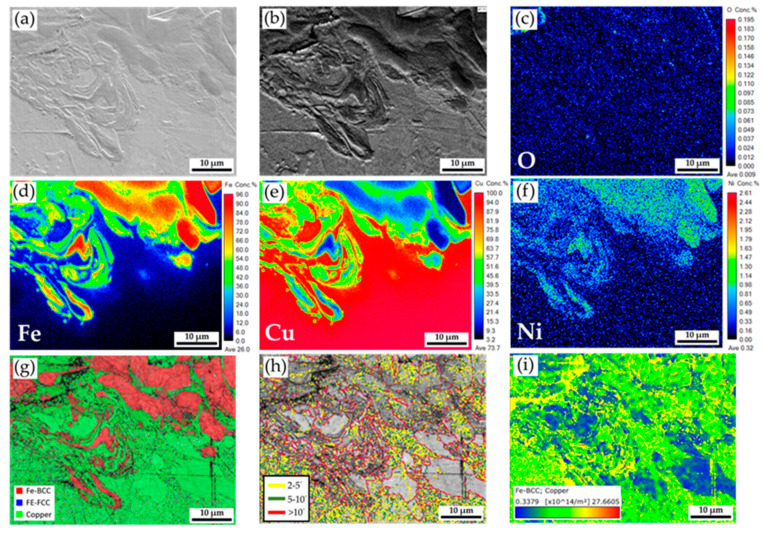
Higher magnification of the area in Figure 4b: (**a**,**b**) EPMA SEI and BEI; (**c**–**f**) EPMA quantitative element mappings of O, Fe, Cu, and Ni in wt%; EBSD (**g**) phase map, (**h**) boundary misorientation map, and (**i**) GND density map.

Figure 5 displays a higher magnification of the red frame area in Figure 4b. Figure 5a,b shows the SEI and BEI. The fusion boundary between the Ni-plated steel and Cu sheets is illustrated in this figure, and the mixture of the steel and Cu is observed. The oxidation close to the weld fusion line is trivial, as demonstrated in Figure 5c. According to the quantitative element mappings of Fe, Cu, and Ni as illustrated in Figure 5d–f, the plated Ni layer on the steel plate is completely dissolved into the melt in the fusion zone of the weld, and Cu and Fe are well mixed near the fusion boundary. The trace of melting convection during the conduction mode of the coaxial laser welding is illustrated in Figure 5d–f. The phase map in Figure 5d also denotes the red BCC Fe and green Cu mixture. The red BCC Fe stands for the ferrite in the steel plate. Figure 5h,i displays the boundary misorientation and GND density maps. It is noted that many grains in this area are characterized by a large number of sub-boundaries and high dislocation density due to the high residual stress after solidification during welding.

### 3.3. Microstructural Analyses of the Fiber/Blue-Light Coaxial Laser Continuous Welding Specimen

**Figure 6:**
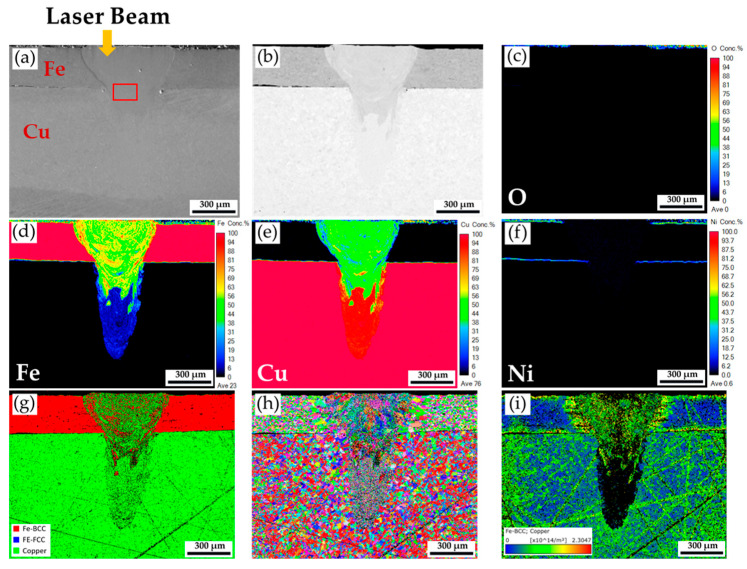
The cross-section of the fiber/blue-light coaxial laser continuous welding specimen: (**a**,**b**) EPMA SEI and BEI; (**c**–**f**) EPMA quantitative element mappings of O, Fe, Cu, and Ni in wt%; EBSD (**g**) phase map, (**h**) IPF, and (**i**) GND density map.

Figure 6 shows the cross-section of the fiber/blue-light coaxial laser beam continuous welding specimen. Figure 6a shows the SEI, and Figure 6b shows the BEI. The fusion zone of the weld is in good shape and free of any defects, e.g., underfill, cracks, and voids, indicating that the energy density of the coaxial laser beam is appropriate. The maximum width of the fusion zone is 606 µm, and the penetration depth is 955 µm. The continuous fiber/blue-light coaxial laser beam welding achieves a keyhole mode fusion zone.

Figure 6c–f shows quantitative O, Fe, Cu, and Ni mappings. The weld is free of oxygen contamination, as demonstrated in Figure 6c. The Ni-plated layer on the steel plate is completely dissolved in the weld fusion zone, as shown in Figure 6f. The morphology of the keyhole mode weld fusion zone (Figure 6a,b,d) is quite different from that of the conduction mode zone (Figure 4a,b,d). In the keyhole mode weld fusion zone, the Fe in the upper sheet penetrates a much deeper depth of the bottom Cu sheet, driven by the convection induced by the fiber/blue-light coaxial laser beam. Integrating the blue-light laser into the coaxial laser beam significantly improves the bottom Cu sheet’s absorption coefficient and demonstrates great application potential.

Figure 6g displays an EBSD phase map of the weld. The phase distribution in the keyhole mode fusion zone (Figure 6g) is very different from that in the conduction mode fusion zone (Figure 4g). In Figure 6g, red BCC Fe (ferrite) and green Cu are mixed in the upper portion of the fusion zone due to the strong convection of metal vapor and plasma in the keyhole mode welding. Fine grains are observed in the IPF illustrated in Figure 6h. The cellular grains in the upper portion of the conduction mode fusion zone, displayed in Figure 4h, are no longer observed in the keyhole mode fusion zone. The plasma convection of the weld pool in the keyhole mode of coaxial laser welding is much stronger than the melt convection of the fusion zone in the conduction mode of welding. Because Cu and Fe are not completely soluble, the phase separation of the red ferrite and green Cu from plasma and vapor is achieved in the fusion zone after the coaxial laser welding, as demonstrated by Figure 6g [37,38].

It is worth mentioning that grain coarsening of the heat-affected zone (HAZ) of the steel and Cu plates is observed in Figure 6h due to the high laser welding heat flux dissipating from the weld fusion zone into the base metals, steel, and Cu plates. The coaxial laser beam irradiates the top Ni-plated steel, which has high absorption coefficients for fiber and blue-light lasers. The heat flux imposed on the top Ni-plated steel is much greater than on the bottom Cu sheet. Therefore, the degree of grain coarsening in the upper steel sheet is higher than in the bottom Cu sheet. Figure 6i shows the EBSD GND density map. It is reasonable that most of the residual thermal stresses after laser welding are localized in the HAZ of the upper steel sheet.

**Figure 7:**
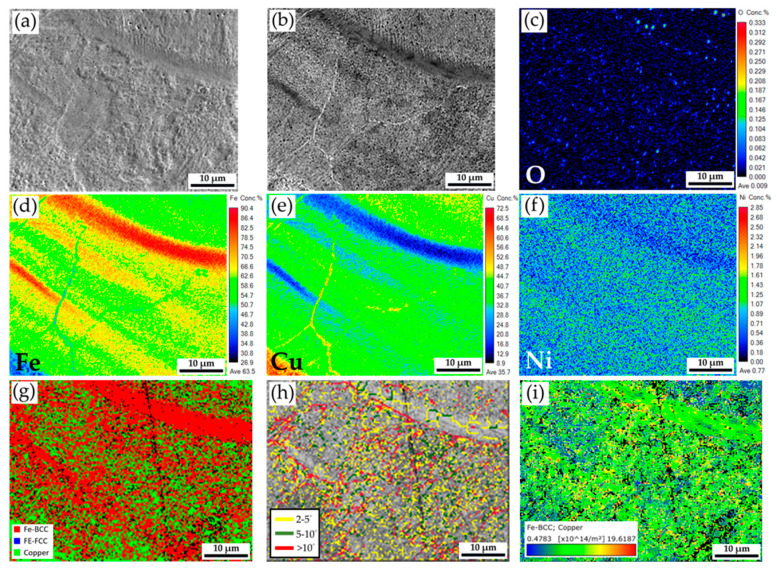
Higher magnification of the area in Figure 6a: (**a**,**b**) EPMA SEI and BEI; (**c**–**f**) EPMA quantitative element mappings of O, Fe, Cu, and Ni in wt%; EBSD (**g**) phase map, (**h**) boundary map, and (**i**) GND density map.

Figure 7a,b shows SEI and BEI at a higher magnification of the red area in the fusion zone shown in Figure 6a, and there is no solidification void in this figure. The oxygen concentration is low, as demonstrated in the quantitative oxygen mapping shown in Figure 7c. It is confirmed that the Ar protection is good enough during fiber/blue-light coaxial laser continuous welding. According to the quantitative Fe, Cu, and Ni mapping results of Figure 7d–f, Fe and Cu are not evenly mixed, and Ni is dissolved into Fe and Cu. Incorporating the phase map in Figure 7g and Fe/Cu mappings in Figure 7d,e, the strip-like red ferrite and the mixture of tiny green Cu droplets in the red ferrite matrix are observed. The microstructure illustrated in the fusion zone does not result from a traditional solidification process because the microstructure is free of cellular or dendritic features. Condensation of the Cu vapor and plasma into tiny Cu droplets in the Fe-rich matrix better explains the microstructure in the fusion zone. Figure 7i shows the GND density map at a higher magnification. The dislocation density of the Fe-rich matrix is higher than that of the Cu droplet, and most residual thermal stresses are in the ferrite of the fusion zone after coaxial laser welding.

### 3.4. The Advantages of the Coaxial Laser Beam in Lap Welding the Nickel-Plated Steel and Copper Sheets

Because the lap joint of welding nickel-plated steel and copper sheets serves as the negative electrode grounding in the mass production of cylindrical lithium-ion batteries, the major issues with the joint are the shape of the fusion zone, phase distribution, and integrity of the weld. The mechanical properties, e.g., microhardness and strength of the weld, are not considered in this study. The integrity of the weld is the most crucial consideration for conducting high-power current in the battery.

Since the resistance spot welding is used in the mass production of cylindrical lithium batteries, laser spot welding was first tested. The YAG laser with a wavelength of 1064 nm is a popular one in the industry, and it has been tested for a long time in the laboratory. Unfortunately, none of the test parameters, including laser power, dwell time, and depth of focus, can successfully weld the bottom copper sheet with an acceptable fusion zone of good integrity. Section 3.1 shows one example to illustrate the weld’s microstructure. In Figure 3, the underfill on the top of the weld demonstrates that increasing the YAG laser power results in the vaporization of the nickel-plated steel. However, there is a trivial contribution to welding the bottom copper sheet due to the low absorption coefficient of the YAG laser beam to copper.

Many applications of the blue-light laser are available in the literature [39,40,41]. Using the blue-light laser for copper welding is not new, and extensive literature is available [31,32,34,36]. However, the current case has not reported the lap laser welding from the nickel-plated steel to the copper side. The concept of a fiber/blue-light coaxial laser beam is introduced to evaluate the feasibility of incorporating a cylindrical lithium battery mass production line. The fiber laser beam can penetrate the nickel-plated steel sheet, and the blue-light laser beam can melt the bottom copper sheet. The coaxial laser spot welding greatly improves the formation of a fusion zone with a mixture of Cu and Fe on the bottom copper sheet, as illustrated in Figure 4 and Figure 5. Introducing the blue-light laser to the coaxial laser beams effectively overcame the high reflectivity of the bottom copper sheet. It successfully formed a lap weld between the nickel-plated steel and copper sheets.

The weld size in the coaxial laser spot welding is much less than that of traditional resistance spot welding. Continuous coaxial laser welding was applied to increase the area of the current pathway in the high-energy-density battery. The fusion zone of the weld is of good integrity and defect-free, as displayed in Figure 6 and Figure 7. It shows potential in the mass production of the next generation of lithium batteries.

## 4. Conclusions

The heterogeneous lap welding of nickel-plated steel and Cu sheets has been investigated. Quantitative chemical and crystallographic analyses of the cross-section of the welded part were examined using EPMA and EBSD. Important conclusions are listed below:

1. The YAG laser beam only penetrates the upper Ni-plated steel sheet and cannot weld the bottom Cu sheet due to the extremely low absorption coefficient of the YAG laser beam. The laser beam has little effect on melting Cu, and only stratification occurs at the interfacial gap between the upper Ni-plated steel and the bottom Cu plate. The YAG laser is inappropriate for welding Ni-plated steel and Cu sheets.
2. Introducing the blue-light laser into the coaxial laser beam significantly improves the spot welding specimen’s quality due to the blue-light laser’s high absorption coefficient by the bottom copper sheet. The fiber laser beam can penetrate the nickel-plated steel sheet, and the blue-light laser beam can melt the bottom copper sheet. The plated nickel layer on the steel sheet is completely dissolved into the weld fusion zone, and a mixture of Fe and Cu is achieved in the weld.
3. Introducing the blue-light laser to the coaxial laser beams overcame the reflectivity of the bottom copper sheet. The fiber/blue-light coaxial laser continuous welding can achieve the best integrity and defect-free welding. It shows potential in the mass production of the next generation of lithium batteries.

## Figures and Tables

**Table 1:** Laser welding conditions used in the experiment.

Type of Laser Welding	Power (W)	Pulse Width (ms)	Spot Size (μm)	Travel Speed (mm/s)
YAG laser spot welding	500	12	150	---
Fiber/blue-light coaxial laser spot welding	1010 (fiber laser) 320 (blue-light laser)	20 20	50 300	---
Fiber/blue-light coaxial laser continuous welding	740 (fiber laser) 320 (blue-light laser)	20 20	50 300	50 50

## Data Availability

The raw data supporting the conclusions of this article will be made available by the authors on request.
